# Murine gammaherpesvirus infection is skewed toward Igλ+ B cells expressing a specific heavy chain V-segment

**DOI:** 10.1371/journal.ppat.1008438

**Published:** 2020-04-30

**Authors:** Christopher M. Collins, Christopher D. Scharer, Thomas J. Murphy, Jeremy M. Boss, Samuel H. Speck

**Affiliations:** 1 Emory Vaccine Center and Department of Microbiology and Immunology, Emory University School of Medicine, Atlanta, Georgia, United States of America; 2 Department of Pharmacology and Chemical Biology, Emory University School of Medicine, Atlanta, Georgia, United States of America; University of North Carolina at Chapel Hill, UNITED STATES

## Abstract

One of the defining characteristics of the B cell receptor (BCR) is the extensive diversity in the repertoire of immunoglobulin genes that make up the BCR, resulting in broad range of specificity. Gammaherpesviruses are B lymphotropic viruses that establish life-long infection in B cells, and although the B cell receptor plays a central role in B cell biology, very little is known about the immunoglobulin repertoire of gammaherpesvirus infected cells. To begin to characterize the Ig genes expressed by murine gammaherpesvirus 68 (MHV68) infected cells, we utilized single cell sorting to sequence and clone the Ig variable regions of infected germinal center (GC) B cells and plasma cells. We show that MHV68 infection is biased towards cells that express the Igλ light chain along with a single heavy chain variable gene, IGHV10-1*01. This population arises through clonal expansion but is not viral antigen specific. Furthermore, we show that class-switching in MHV68 infected cells differs from that of uninfected cells. Fewer infected GC B cells are class-switched compared to uninfected GC B cells, while more infected plasma cells are class-switched compared to uninfected plasma cells. Additionally, although they are germinal center derived, the majority of class switched plasma cells display no somatic hypermutation regardless of infection status. Taken together, these data indicate that selection of infected B cells with a specific BCR, as well as virus mediated manipulation of class switching and somatic hypermutation, are critical aspects in establishing life-long gammaherpesvirus infection.

## Author summary

Murine gammaherpesvirus 68 is a rodent pathogen that is closely related to the human gammaherpesviruses Epstein-Barr virus and Kaposi’s sarcoma-associated virus. All know gammaherpesviruses are associated with the development of lymphomas, as well as other cancers, in a small subset of infected individuals–particularly those with underlying defects in their immune system (i.e., transplant recipients and HIV infected patients). Because there are very limited small animal models for the human gammaherpesviruses, studies on murine gammaherepsviruses 68 can provide important insights into critical aspects of gammaherpesvirus infections and the association of these viruses with disease development. Another feature of all gammaherpesviruses is their ability to establish a chronic infection of their host–where the virus is maintained for the lifetime of the infected individual. The major target cell harboring chronic gammaherepsvirus infection are B lymphocytes–the cells in the immune system that produce antibodies in response to infections. Here we provide a detailed characterization of the populations of B lymphocytes that become infected by murine gammaherpesvirus 68. This has led to the identification of a specific population of B lymphocytes that is preferentially infected by the virus. This supports a model in which murine gammaherpesvirus infection of B lymphocytes is not random. However, it remains unclear why the virus targets this specific population of B cells for infection.

## Introduction

One of the defining characteristics of the human gammaherpesviruses Epstein-Barr virus (EBV) and Human herpesvirus 8 (HHV-8 also known as Kaposi’s sarcoma associated herpesvirus or KSHV) is their ability to establish life-long infection in memory B cells. Murine gammaherpesvirus 68 (MHV68) also establishes life-long infection in B cells [1, 2]. At the peak of infection, the majority of MHV68 infected cells have a germinal center (GC) B cell phenotype [3–7], with the remaining infected cell population consisting largely of plasma cells [4, 8]. In establishing latent infection of B cells, MHV68 takes advantage of GC B cell proliferation during the germinal center response to virus infection resulting in the expansion of the pool of latently infected cells [9]. Notably, differentiation of infected B cells to plasma cells has been shown to induce viral reactivation [8].

In a T cell dependent GC reaction, B cells undergo selection for cells whose B cell receptors (BCR) have high affinity for antigen [10]. These GC B cells undergo iterative cycles of proliferation and somatic hypermutation (SHM) as centroblasts in the dark zone of the germinal center followed by differentiation to centrocytes. These centrocytes take up antigen through their BCR from follicular dendritic cells in the light zone of the germinal center and present it on MHC II to cognate T follicular helper (T_FH_) cells, which in turn provide survival and proliferation signals. T_FH_ cells are limiting, and B cells whose BCRs have high affinity for antigen are able to out-compete those with lower affinities, resulting in selection of cells with high affinity for antigen. Surviving B cells can then exit the germinal center reaction and persist as either memory B cells or long-lived plasma cells. Because MHV68 infects both GC B cells and plasma cells during establishment of latency, this suggests that infected cells may undergo some form of selection. Indeed, it has been shown that establishment of latent infection in B cells by MHV68 is not stochastic [11].

The BCR is composed of two heavy chains and two light chains, as well as two signaling subunits, CD79a and CD79b [12]. The antigen binding domain of the BCR consists of variable (V), diversity (D) and joining (J) gene segments in the heavy chain and V and J gene segments in the light chain. There are 110 functional variable heavy chain (VH) genes in C57Bl6 mice that are grouped into 16 different families [13], 10 D genes grouped into 4 families [14] and 4 JH genes [15]. Ig light chains are encoded by two different loci termed Ig kappa (Igκ) and Ig lambda (Igλ). In the Igκ locus, there are 95 functional Vκ genes that are grouped into 19 families [16], and 4 Jκ genes [17]. The Igλ locus is much less diverse, containing only 3 functional Vλ and 3 functional Jλ genes, and as a result, only around 10% of murine B cells express a Igλ light chain [18, 19].

Although immunoglobulins expressed by B cells play a central role in their fate, very little is known about the immunoglobulin genes expressed by cells infected with MHV68. For EBV, it has been shown that the distribution of isotypes and variable heavy chain usage of infected cells does not differ significantly from uninfected cells [20]. Additionally, although a subset of infected cells display auto-reactive or poly-reactive BCRs, the frequency of these cells among the total infected memory B cell compartment is lower than that found in the uninfected population [20]. Little is known about the specificity and BCR repertoire of KSHV infected B cells, but it has been shown that KSHV preferentially infects tonsillar B cells that express a lambda light chain *in vitro* [21]. Additionally, in Multicentric Castleman’s disease, KSHV infected cells are restricted to cells expressing the Igλ light chain [22–24], suggesting there may be unexplored relationships between the BCR and viral infection.

In this study, we utilized single cell sorting to isolate MHV68 infected GC B cells and plasma cells in order to characterize their immunoglobulin genes. We show that infected cells are biased towards cells that express an Igλ light chain along with the VH gene IGHV10-1*01. This biased population results from the clonal expansion of these cells but is not viral antigen specific, as antibodies cloned from these cells do not react to viral antigens. Additionally, the isotypes of MHV68 infected GC B cells and plasma cells are significantly different from uninfected cells, with infected GC B cells more likely to be IgM+, whereas infected plasma cells are more likely to be class-switched to IgG. Furthermore, both uninfected and infected class-switched plasma cells show little somatic hypermutation, with the majority of both populations completely lacking any mutations in their heavy chain variable genes. Taken together, these analyses provide important insights that suggest that MHV68 manipulates B cell biology to establish life-long infection.

## Results

### MHV68 infection is skewed towards B cells that express an Igλ light chain

**Fig 1:**
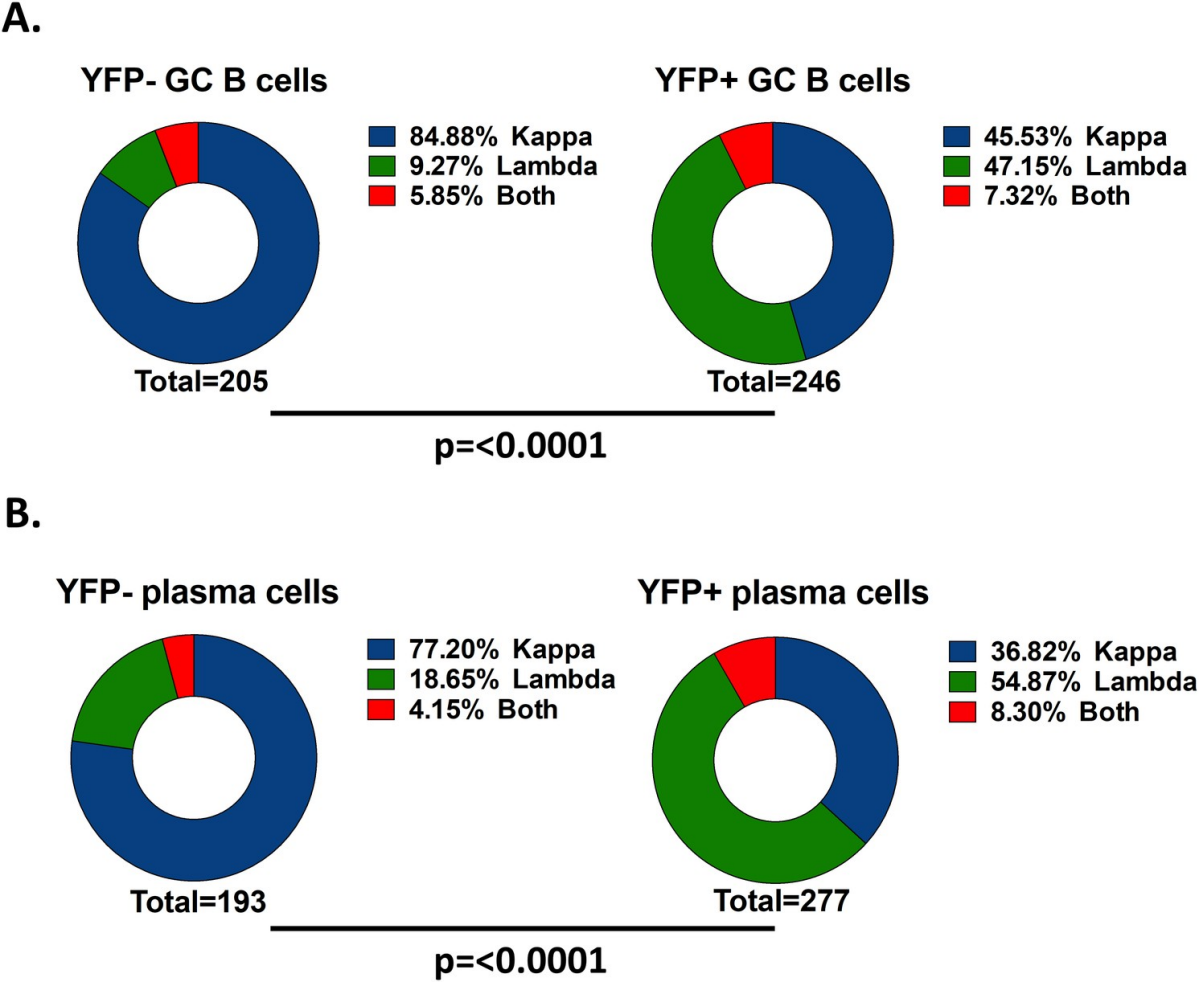
MHV68 infected cells are biased towards cells expressing the Igλ light chain. Mice were infected intranasally with 1,000 pfu of MHV68-H2bYFP and splenocytes were harvested 18 days post-infection. Cells from the indicated populations were single cell sorted, and cDNA was prepared from RNA from single cells and used as template to PCR amplify the variable regions of the immunoglobulin heavy and light chains. Shown is the percentage of cells from each population that expressed either the Igκ, Igλ or both light chains from (A) germinal center B cells and (B) plasma cells. p values were calculated by Chi square test. Data are pooled from 5 mice per population.

To begin characterizing the immunoglobulin repertoire of MHV68 infected B cells and plasma cells, we utilized a previously described strategy to identify and clone the heavy and light chain variable regions of murine immunoglobulin genes from single cells [25]. Using this strategy, we performed single cell sorting to purify MHV68 infected and uninfected GC B cells, as well as infected and uninfected plasma cells, from mice infected with a recombinant MHV68 that expresses YFP from a neutral locus within the viral genome (MHV68-H2bYFP) [3]. Cells were sorted based on YFP expression and surface marker phenotype (Table 1). During the initial PCR reaction to amplify the variable regions, the vast majority of uninfected cells (YFP-)from both GC (Fig 1A, S1 Table) and plasma cell populations (Fig 1B, S1 Table) were positive for the Igκ chain, albeit at slightly lower levels than the frequency of ca. 90% seen in naïve B cells. In stark contrast, MHV68 infection (YFP+ cells) was heavily biased towards cells that expressed an Igλ light chain (Fig 1, S1 Table). Nearly half of the PCR reactions from YFP+ GC B cells were positive for the lambda chain (Fig 1A, S1 Table), whereas over half of the YFP+ plasma cells were Igλ+ (Fig 1B, S1 Table). Taken together, these data indicate that infection of GC B cells and plasma cells by MHV68 is not random, and that there is selection at some level among MHV68 infected cells for those that express the Igλ light chain.

**Table 1:** 

Cell type	Phenotype
Uninfected GC B cells	YFP^-^, B220^-^, CD95^+^, GL7^high^
MHV68Y2bYFP infected GC B cells	YFP^+^, B220^-^, CD95^+^, GL7^high^
Uninfected plasma cells	YFP^-^, B220^lo-neg^,CD138^+^
MHV68H2bYFP infected plasma cells	YFP^+^, B220^lo-neg^,CD138^+^

### MHV68 infected Igλ expressing cells are biased towards cells that express a single heavy chain variable gene

**Fig 2:**
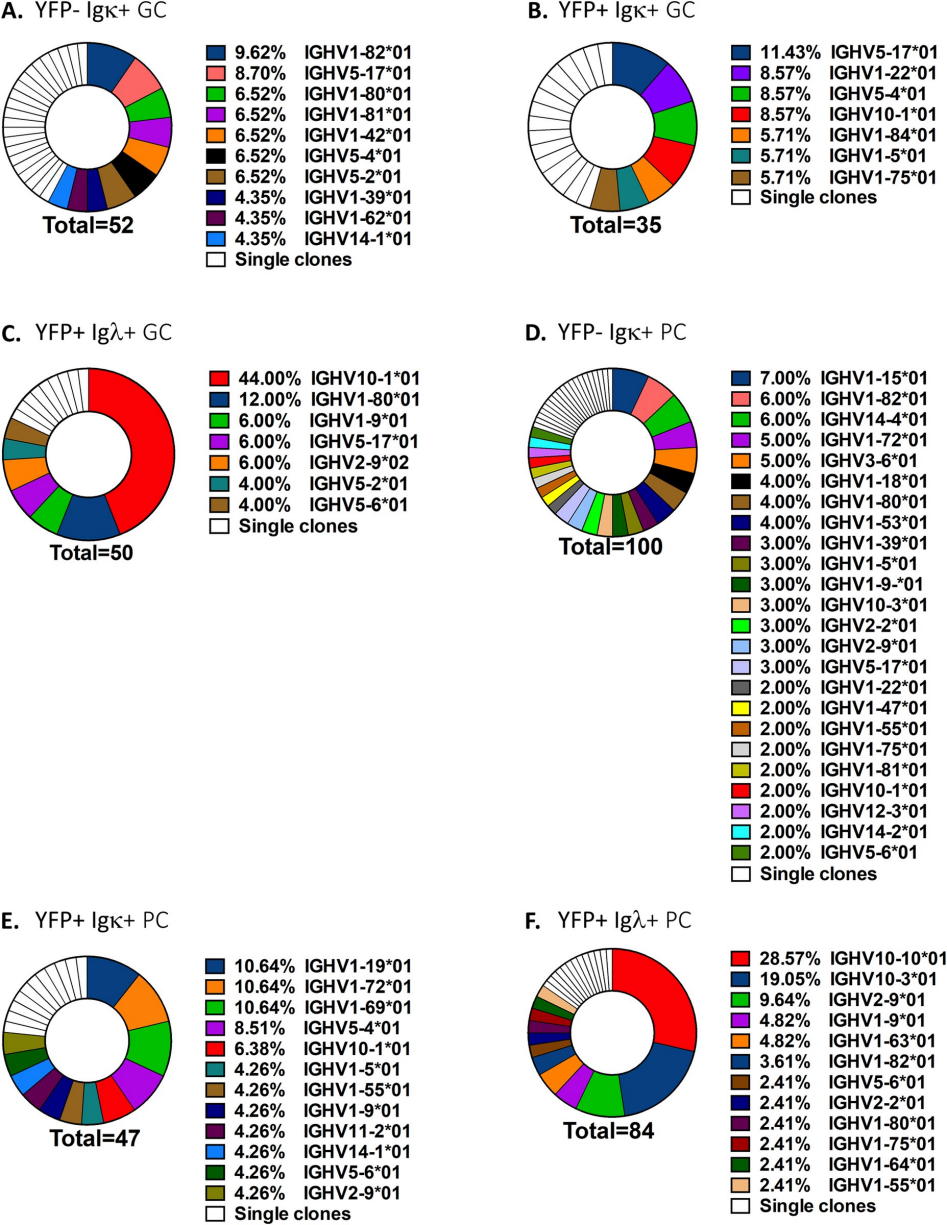
MHV68 infected cells that express the Igλ+ light chain are biased towards cells that express a common heavy chain variable gene. To determine if VH gene usage is biased among the sorted populations, VH genes from sorted single cells were identified by Ig BLAST. Shown are the frequencies of each VH gene within the indicated populations. IGHV genes represented by single clones are shown in white. Data are pooled from 4–5 mice per population.

To further analyze the variable region genes, the variable regions from single cells in which both the heavy and light chain PCR reactions yielded products were sequenced. In both the uninfected (YFP-) Igκ+ and infected (YFP+) Igκ+ GC B cell populations, there was no dominant VH gene represented (Fig 2A and 2B). There were 10 different VH genes present between 4.35–9.62% of the total populations in YFP- Igκ+ GC B cells, with the remaining VH genes represented by single clones (Fig 2A). In the YFP+ Igκ+ GC B cell population there were 7 different VH genes that were present at frequencies between 5.7–11.4% (Fig 2B). However, the infected (YFP+) Igλ+ GC B cell population was biased towards cells that expressed a single heavy chain variable gene, as 44% of the cells in this population expressed IGHV10-1*01 (Fig 2C). Similarly, there was no dominant VH segment used in the either the uninfected (YFP-) Igκ+ or infected (YFP+) Igκ+ plasma cell populations (Fig 2D and 2E), but the infected (YFP+) Igλ+ plasma cell population showed a similar enrichment in that 28.6% of this population expressed IGHV10-1*01 (Fig 2D). Additionally, 19% of YFP+ Igλ+ plasma cells expressed IGHV10-3*01, which is in the same gene family as IGHV10-1*01 and shares 97% similarity with it. A rigorous analysis of the uninfected (YFP-) Igλ+ fraction was not possible due to the paucity of these cells. However, a limited analysis revealed that in the uninfected (YFP-) GC population 1of 5 Igλ+ cells whose variable regions were sequenced expressed IGHV10-1*01, while in the uninfected (YFP-) plasma cell population 7of 20 Igλ+ cells sequenced expressed IGHV10-1*01. Thus, like the infected Igλ+ GC and plasma cell populations, the uninfected (YFP-) Igλ+ populations are also enriched for IGHV10-1*01 expression. This indicates that, while MHV68 infection is biased towards lambda expressing B cells, there may not be any further selection for Ig heavy chain variable region expression.

**Fig 3:**
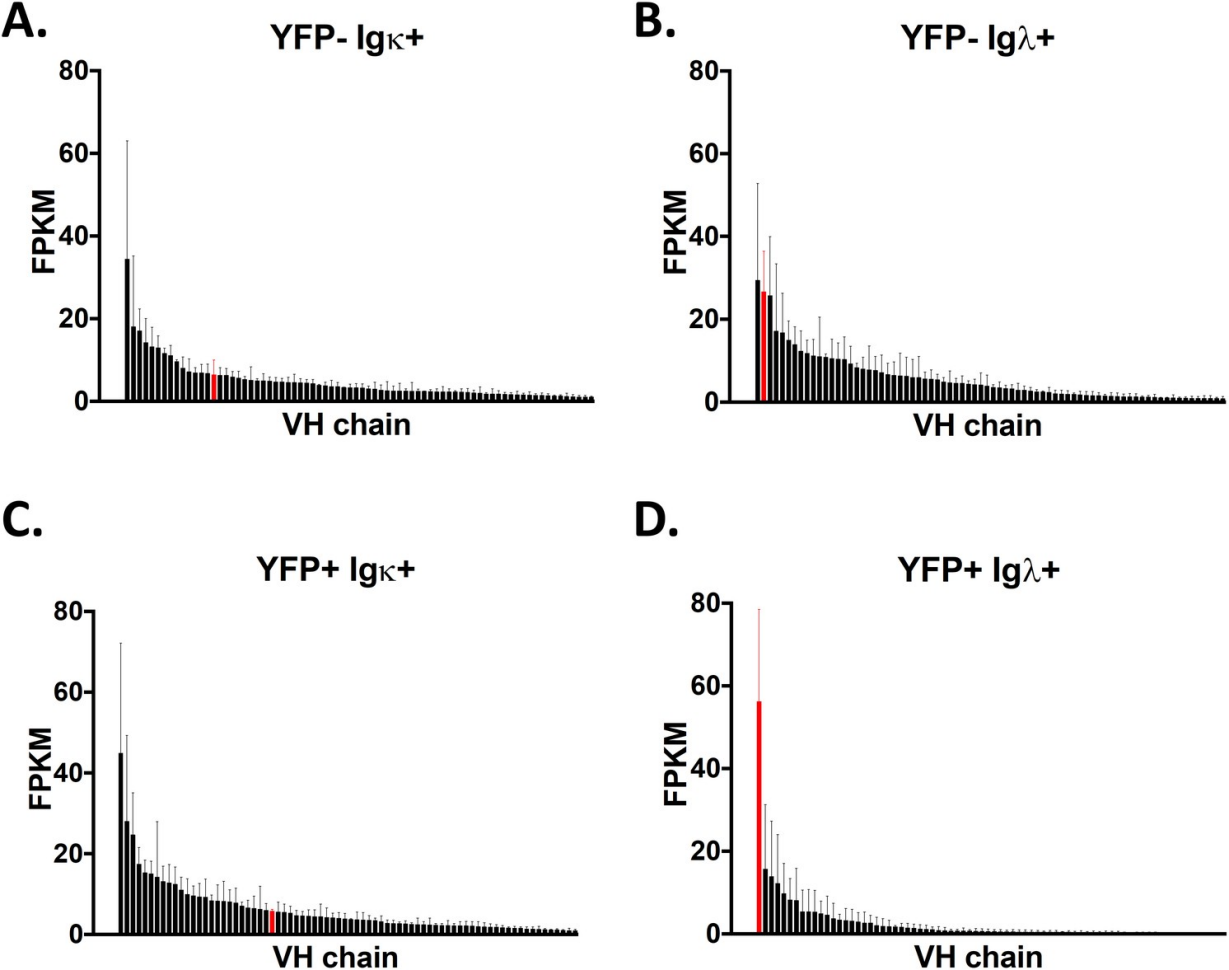
Rank order of VH chains from the indicated populations. Mice were infected intranasally with 1,000 pfu of MHV68-H2bYFP, and splenocytes were harvested 18 days post-infection. Cells from the indicated populations were sorted and RNA sequencing was performed to determine the relative levels of expression of each VH gene. Shown are the 75 most highly expressed VH gene from each population, with IGHV10-1*01 shown in red in each population. Transcript levels were normalized to fragments per kilobase per million reads (FPKM).

To confirm these results and better address the latter issue, RNA-seq analysis was performed on Igκ+ and Igλ+ cells sorted from both uninfected (YFP-) and MHV68 infected (YFP+) GC B cells populations and the expression of Ig segments was annotated. Because plasma cells do not express surface Ig, sorting was limited to GC B cells. There was a wide range of heavy chains used in both the uninfected (YFP-) populations, as well as in the infected (YFP+) Ig+ population (Fig 3A–3C). Although some heavy chains appeared to be more abundant, in each case this was due to unusually high expression in 1 of the 4 replicates sequenced in each population. However, in the MHV68 infected (YFP+) Igλ+ population, overall heavy chain usage was less varied, and IGHV10-1*01 was the most highly expressed VH gene in each replicate (Fig 3D). In agreement with heavy chain usage data for the few YFP- Igλ+ GC B cells we were able to purify by single cell sorting and sequence, IGHV0-1*01 was also present at high levels in the purified YFP- Igλ+ population, but was not the dominant VH gene expressed. This more rigorous analysis indicates that MHV68 infection also selects for lambda light chain positive cells that express the IGHV10-1*01 heavy chain variable region.

### The MHV68 infected Igλ+ IGHV10-1*01 population arises through clonal expansion

The most likely explanation for the over-representation of YFP+ Igλ+ cells that express the IGHV10-1*01 heavy chain is that they arose through clonal expansion. To determine if this was the case, sequences from cells that yielded complete sequences for both the heavy chain and light chain variable regions were analyzed. Clones were defined as two or more cells from the same mouse that shared the same VH gene, D gene, and J gene in the heavy chain and the same VH gene and J gene in the light chain.

**Fig 4:**
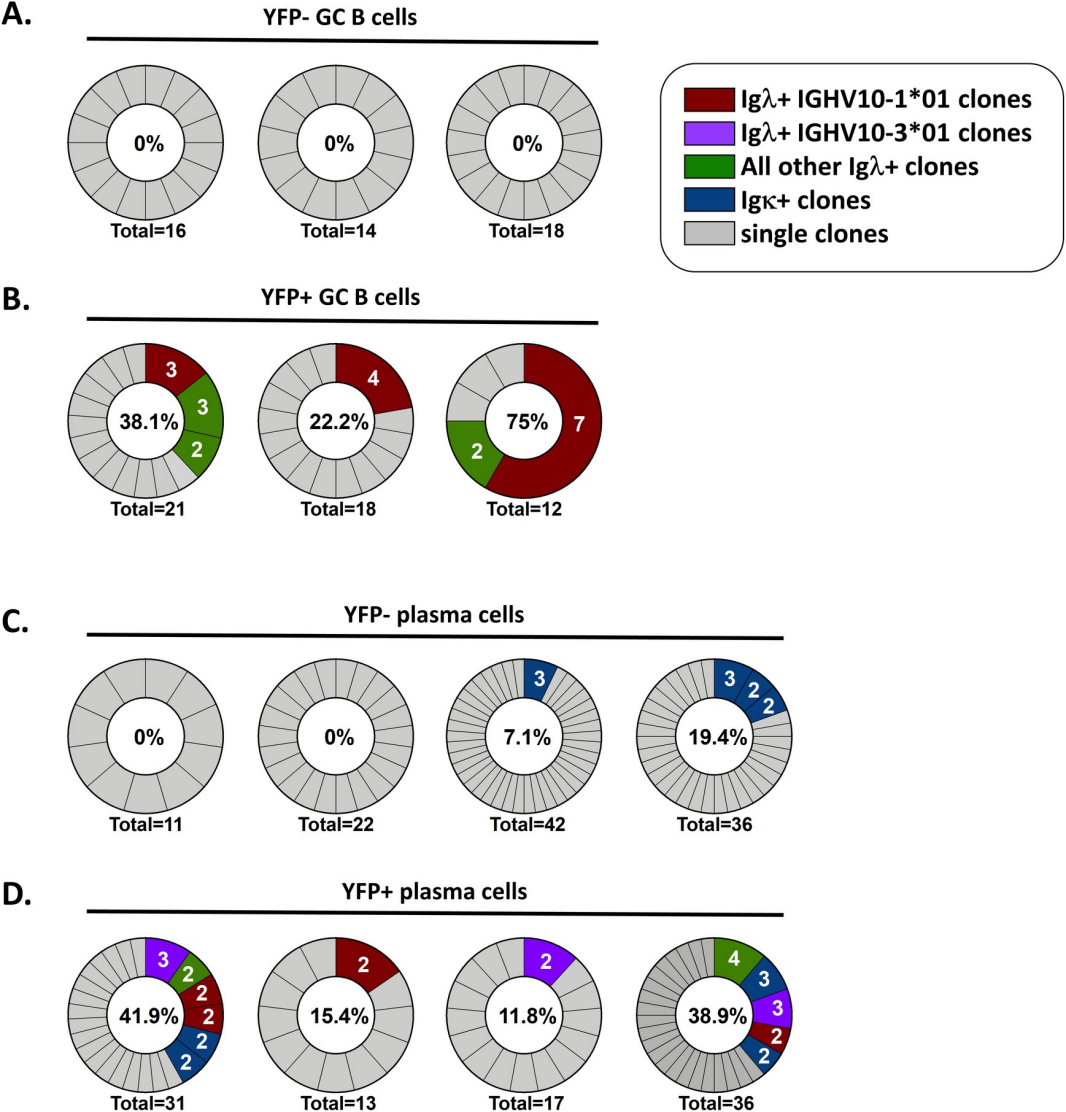
The MHV68 infected Igλ+expressing cell populations arise through clonal expansion. Identification of clonal expansions in each of the indicated populations. Clones were defined as two or more cells from the same mouse that shared the same VDJ rearrangement in the heavy chain and the same VJ rearrangement in the light chain. Each pie chart represents clones from a single mouse. Percentages within each chart are the percentage of cells represented by clonal expansions within the total population. “Igλ+ IGHV10-1*01” and “Igλ+ IGHV10-3*01” represent clonal expansions that consisted of cells that expressed the Igλ light chain and IGHV10-1*01 or IGHV10-3*01 respectively. “All other Igλ clones” represent clonal expansions that expressed an Igλ light chain and a VH gene other than IGHV10-1*01 or IGHV10-3*01. “Igκ+ clones” represent clonal expansions consisting of cells that expressed the Ig kappa light chain, and single clones represent cells whose VDJ rearrangement was represented by one cell. (A) Clonal expansions found within (A) YFP- GC B cells, (B) YFP+ GC B cells, (C) YFP- plasma cells, and (D) YFP+ plasma cells.

In the uninfected GC B cell population, no clonal populations were identified in YFP- cells (Fig 4A). This is probably due to the small sample size, and analysis of a larger set of cells would undoubtedly reveal the presence of clonal expansion. However, in contrast, when a similar number of MHV68 infected (YFP+) GC B cells was analyzed, multiple populations of clonally expanded cells were present (Fig 4B). Notably, all of the clonally expanded populations were found within the in Igλ+ population, including three populations that expressed IGHV10-1*01. As discussed above for the uninfected (YFP-) population, this does not discount clonal expansion of infected (YFP+) Igκ+ cells due to the small sample size but does indicate that clonal expansions in the infected (YFP+) Igλ+ population are more abundant.

In the plasma cell populations, clonal expansions were found in uninfected (YFP-) plasma cells, but were not as frequent as in the infected (YFP+) population (Fig 4C and 4D). The expanded populations in uninfected (YFP-) plasma cells all expressed the Igκ light chain, which is not surprising due to the low frequency of Igλ+ cells in the YFP- fraction. In the MHV68 infected (YFP+) plasma cell population, clonal expansions were found in both the Igκ+ and Igλ+ fractions (Fig 4D). Each of the YFP+ Igκ+ clonal expansions expressed a different VH gene, whereas multiple clonal expansions of YFP+ Igλ+ plasma cells expressed either IGHV10-1*01 or IGHV10-3*01 (Fig 4D). Taken together, these data suggest that the over-represented populations of YFP+ Igλ+ cells arise through clonal expansion.

### MHV68 infected cells differ from uninfected cells in isotype usage

**Fig 5:**
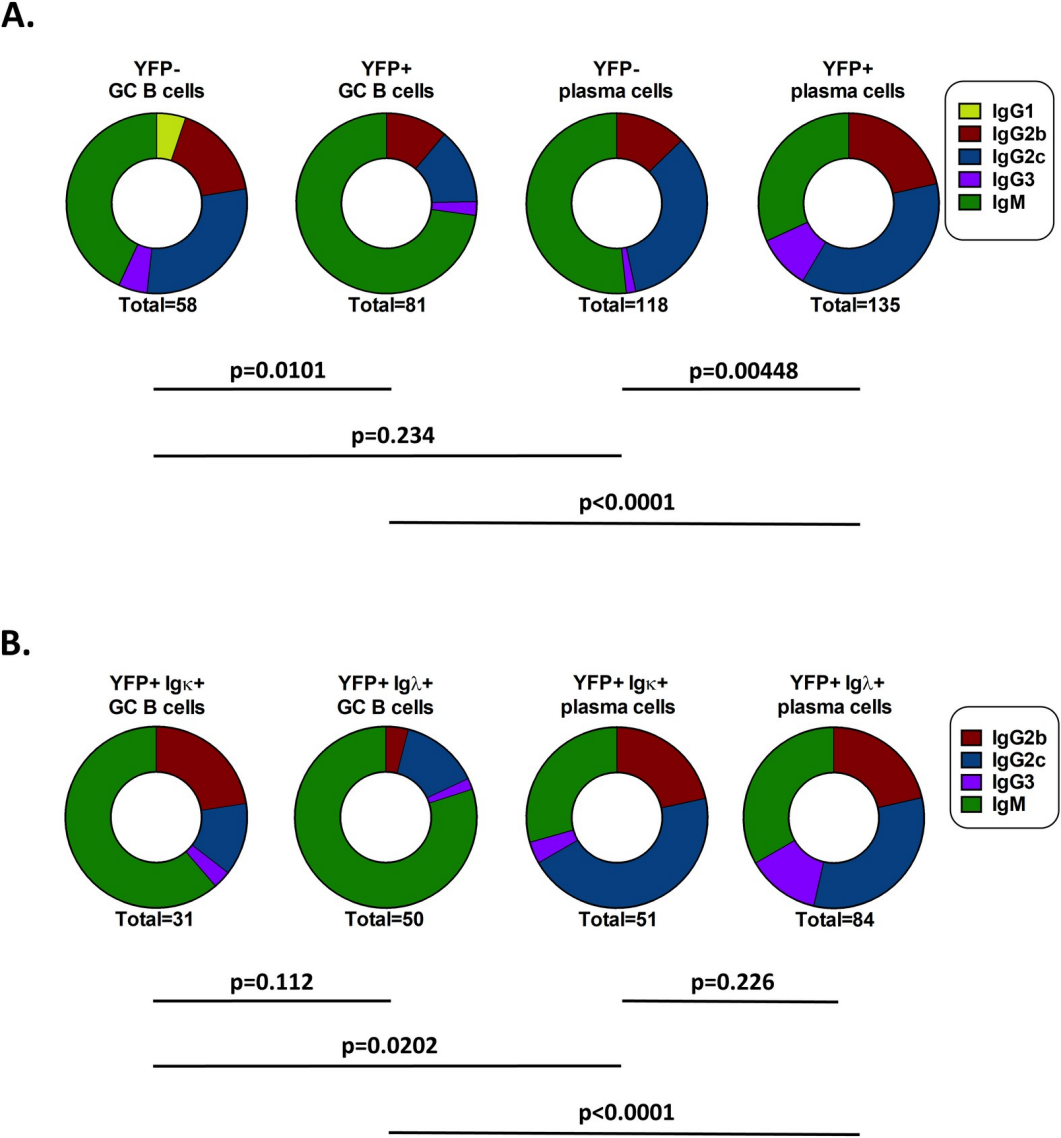
Isotype usage in MHV68 infected cells is significantly different than uninfected cells. The sequences of heavy chain PCR products were analyzed to determine the isotypes of sorted cells. (A) Comparison of YFP- and YFP+ GC B cells and YFP- and YFP+ plasma cells. p values were determined by Fisher’s exact test with Bonferroni correction. (B) Comparison of YFP+ Igκ+ and YFP+ Igλ+ GC B cells and plasma cells. p values were determined by Fisher’s exact test with Holm correction.

It has previously been shown that at early time points during MHV68 infection, the most abundant antibody secreting cells express antibodies of the IgM isotype, followed by IgG2c (labeled IgG2a in that work) and IgG2b [26]. However, little is known about the isotypes of GC B cells or infected plasma cells. To compare the distribution of isotypes among the uninfected (YFP-) and infected (YFP+) GC B cells and plasma cells, the isotypes of each cell from the various populations was identified by sequence analysis. Although there was no significant difference in the distribution of isotypes among uninfected (YFP-) GC B cells and uninfected (YFP-) plasma cells, the distribution of isotypes of infected (YFP+) GC B cells was significantly different than that of infected (YFP+) plasma cells (Fig 5A). This was mainly due to a higher frequency of IgM+ cells in the YFP+ GC B cell population (p<0.0001, Fisher’s exact test) and a higher frequency of IgG2c+ cells in the YFP+ plasma cell population (p = 0.003, Fisher’s exact test). Additionally, the distribution of isotypes between uninfected (YFP-) and infected (YFP+) GC B cells was significantly different (Fig 5A). There was a higher frequency of IgM+ cells in the infected (YFP+) GC B cell population that approached statistical significance (p = 0.059, Fisher’s exact test), but was not statistically significant. The distribution of isotypes in the uninfected (YFP-) plasma cell population was also significantly different than the infected (YFP+) plasma cell population (Fig 5A), mainly due to an increased frequency of IgM+ cells in the uninfected (YFP-) population (p = 0.037, Fisher’s exact test).

These differences in isotype distribution were not due to the over-representation of Igλ+ cells in the infected (YFP+) populations, as there was no difference in isotype usage between Igκ+ and Igλ+ cells in either the infected (YFP+) GC B cell or infected (YFP+) plasma cell populations (Fig 5B). However, the significant difference was maintained between infected (YFP+) GC B cells and infected (YFP+) plasma cells that expressed the same light chain (Fig 5B). Taken together, this indicates that the distribution of isotypes used in MHV68 infected cells is significantly different than that of uninfected cells, and that class-switching in the infected (YFP+) Igλ+ populations is not significantly different than in infected (YFP+) Igκ+ populations.

### MHV68 infection alters somatic hypermutation in class-switched cells

The differences seen in isotype usage were mainly due to differing levels of class-switching, as there was a higher frequency of infected (YFP+) GC B cells that expressed IgM+ than in the uninfected (YFP-) GC B cell population, while fewer infected (YFP+) plasma cells were IgM+ compared to their uninfected (YFP-) counterparts. In the infected (YFP+) GC B cell population, these IgM+ cells may be early GC B cells that have yet to undergo class-switching, and thus would be expected to have little to no somatic hypermutation (SHM). Alternatively, they may be destined to become GC derived IgM+ memory B cells and would be expected to have levels of SHM similar to class-switched GC B cells. In the plasma cell populations, class-switched plasma cells are GC derived, and having traversed the GC reaction would be expected to have relatively high levels of SHM. Non-class switched, IgM+ plasma cells may be either GC derived or of extra-follicular origin. GC derived IgM+ plasma cells would be expected to display similar levels of SHM as class-switched plasma cells, whereas extra-follicular derived plasma cells would not be expected to display SHM.

**Fig 6:**
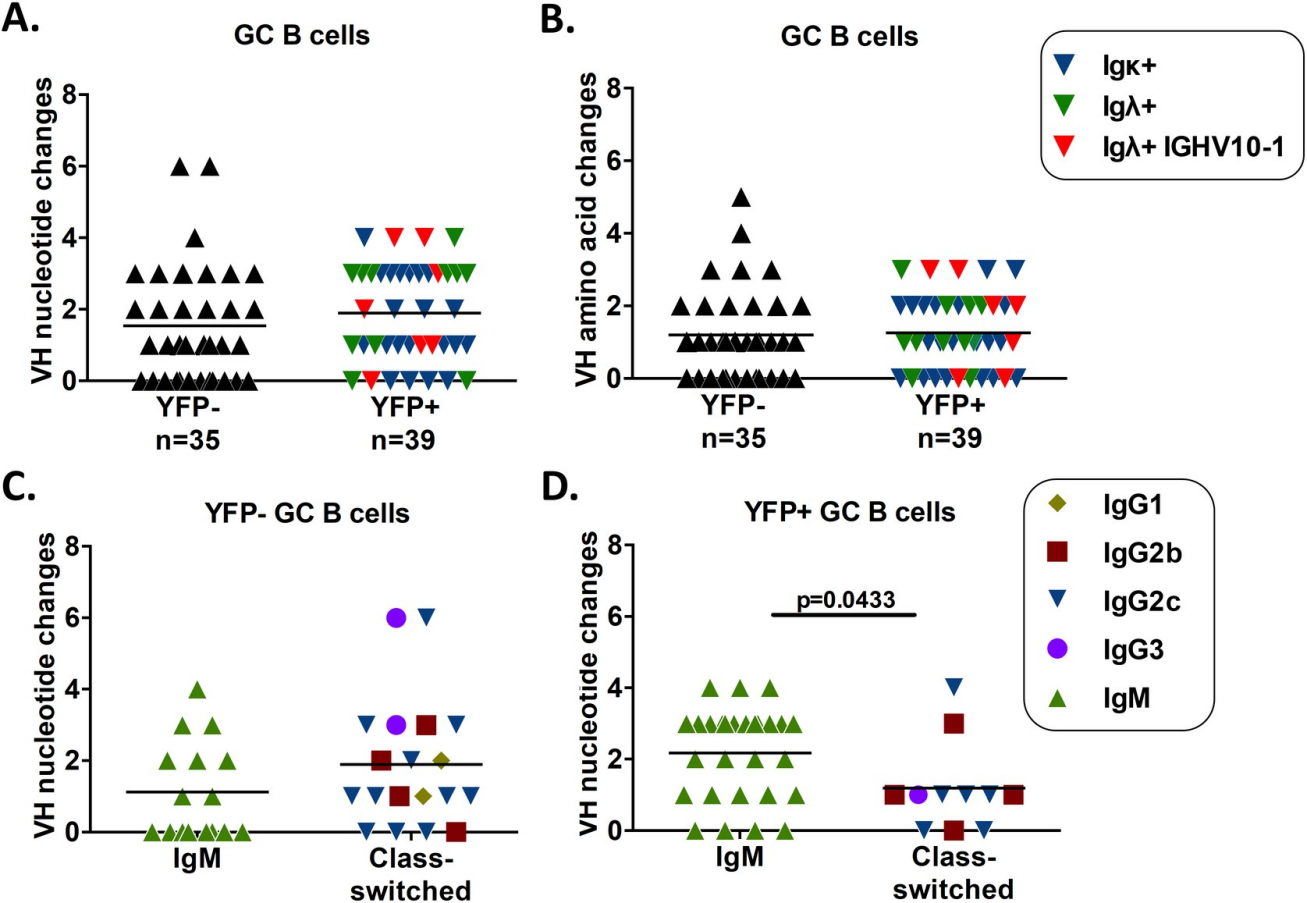
MHV68 infected GC B cells have similar levels of SHM to uninfected cells. Comparison of somatic hypermutation in the Ig heavy chain sequence of germinal center B cells relative to germline sequence. (A) Comparison of the number of nucleotide changes in the VH gene of YFP- and YFP+ GC B cells. (B) Comparison of the number of amino acid changes in the VH gene of YFP- and YFP+ GC B cells. (C) Comparison of the number of nucleotide changes found in IgM+ and class-switched GC B cells in the YFP- population. (D) Comparison of the number of nucleotide changes found in IgM+ and class-switched GC B cells in the YFP+ population. p value was determined by Mann Whitney U test.

Analysis of SHM in VH genes cloned from GC B cells revealed that MHV68 infected GC B cells undergo SHM at a similar rate to uninfected GC B cells (Fig 6A). The average number of mutations in uninfected (YFP-) GC B cells was 1.45, whereas it was slightly higher in infected (YFP+) GC B cells at 1.93, but this difference was not statistically significant. Most of these nucleotide changes resulted in amino acid changes, as 77.8% (42/54) nucleotide changes in uninfected (YFP-) GC B cells resulted in amino acid changes, whereas 66.2% (49/74) mutations resulted in amino acid changes in the infected (YFP+) GC B cell population (Fig 6B). Additionally, SHM in the infected (YFP+) IGHV10-1*01 population was randomly distributed among the range of mutations seen, suggesting that selection due to SHM does not play a significant role in the bias seen in this population. In the uninfected (YFP-) GC B cell population, there was no significant difference in the level of SHM between non-class switched, IgM+ GC B cells and class-switched GC B cells (Fig 6C). Additionally, 50% (8/16) of the IgM+ population displayed SHM, indicating that the uninfected (YFP-) GC B cell population contains cells that may be destined to become IgM+ memory B cells. In the infected (YFP+) GC B cell population, there was a significantly higher level of SHM in the IgM+ population compared to the class-switched population (Fig 6D). The majority of IgM+ GC B cells in the YFP+ population displayed SHM, as 85.6% (24/28) had mutated VH genes (Fig 6D). The increased frequency of IgM+ GC B cells in the infected (YFP+) population (Fig 5A), along with the presence of SHM in the majority of this population (Fig 6D), suggests that a significant fraction of infected GC B cells may be destined to become IgM+ memory B cells.

**Fig 7:**
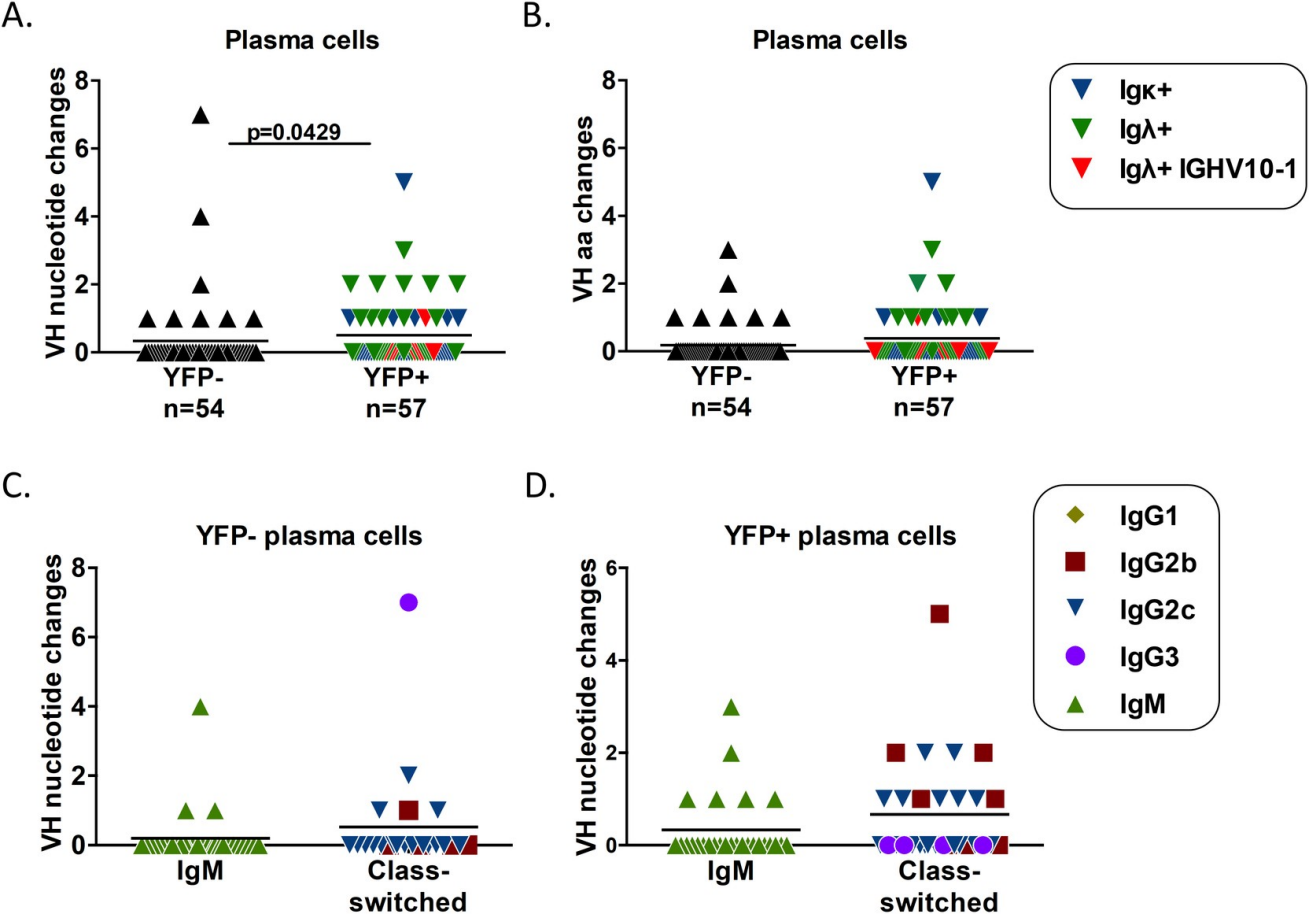
MHV68 infected plasma cells have increased SHM compared to uninfected cells, but SHM is reduced in all class-switched plasma cells. Comparison of somatic hypermutation in the Ig heavy chain sequence of plasma cells relative to germline sequence. (A) Comparison of the number of nucleotide changes in the VH gene of YFP- and YFP+ plasma cells. (B) Comparison of the number of amino acid changes in the VH gene of YFP- and YFP+ plasma cells. (C) Comparison of the number of nucleotide changes found in IgM+ and class-switched plasma cells in the YFP- population. (D) Comparison of the number of nucleotide changes found in IgM+ and class-switched plasma cells in the YFP+ population. p value was determined by Mann Whitney U test.

In the plasma cell populations, there was a significant difference in the level of SHM with the infected (YFP+) plasma cells exhibiting significantly more SHM than the uninfected (YFP-) plasma cell population (Fig 7A). The average number of mutations in the uninfected (YFP-) plasma cell population was 0.33, whereas it was 0.51 in infected (YFP+) plasma cells. Similar to the GC B cell fraction, most of the nucleotide changes resulted in amino acid changes (Fig 7B), as 55.6% (10/18) nt changes in the uninfected (YFP-) plasma cell population resulted in amino acid changes, whereas 75.9% (22/29) of nt changes in the infected (YFP+) plasma cell population resulted in amino acid changes. Additionally, the percentage of infected (YFP+) plasma cells displaying SHM was higher than that of the uninfected (YFP-) fraction, with 31.5% (18/57) of infected (YFP+) plasma cells containing at least 1 nucleotide change, whereas only 14.8% (8/54) of uninfected (YFP-) plasma cells displayed SHM (Fig 7A).

The higher percentage of infected (YFP+) plasma cells that displayed SHM was consistent with the increased percentage of class-switched cells in this population compared to uninfected (YFP-) plasma cells (Fig 5A). However, while the majority of infected (YFP+) plasma cells were class-switched (Fig 5B), only 31.5% displayed SHM, (Fig 7A). This seeming inconsistency was due to very little SHM in class-switched plasma cells, as 60% (18/30) of class-switched infected (YFP+) plasma cells displayed no SHM despite being GC derived (Fig 7D). Similarly, the majority of class-switched uninfected (YFP-) plasma cells also displayed no SHM (Fig 7C). Taken together, these data indicates that infected (YFP+) plasma cells undergo more SHM than uninfected (YFP-) plasma cells, but SHM may not be a reliable indicator of origin for plasma cells during MHV68 infection as most class-switched, germinal center derived plasma cells displayed no SHM.

### The majority of GC B cells and plasma cells induced during MHV68 infection are not viral antigen specific

**Fig 8:**
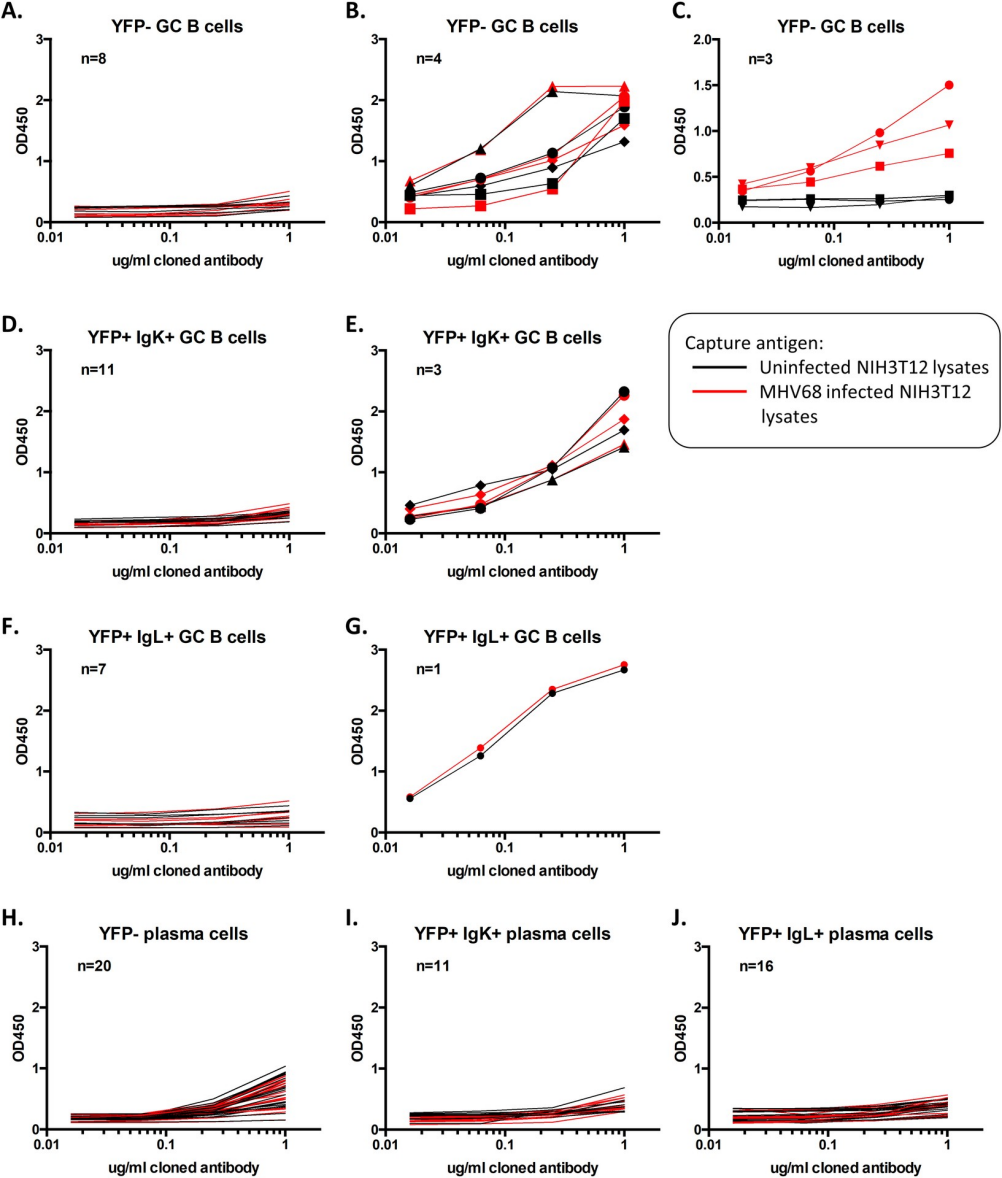
The majority of GC B cells and plasma cells induced during MHV68 infection are not viral antigen specific. Cloned antibodies were tested for reactivity to viral antigens by ELISA. Plates were coated with lysates from either uninfected NIH3T12 cells (black lines) or with lysates from MHV68-H2bYFP infected cells (red lines), and four-fold serial dilutions of each cloned antibody, starting with 1ug, were tested.

Since BCR specificity plays a crucial role in selection of GC B cells and plasma cells, we assessed whether this played a role in the bias seen in MHV68 infected cells. To do this, the variable regions of the heavy and light chains were cloned into expression vectors that contained the hIgG1, Igκ, or Igλ constant regions as previously described [25]. Reactivity was tested by ELISA, comparing reactivity to lysates prepared from NIH3T12 cells infected with MHV68 to that of lysates from uninfected NIH3T12 cells. The reactivity of antibodies cloned from uninfected (YFP-) GC B cells fell into three patterns of reactivity. The majority of antibodies were non-reactive, as they did not react with either lysate (Fig 8A). Others appeared to be autoreactive, as they reacted equally to lysates from both uninfected and infected cells (Fig 8B), while the remaining antibodies were clearly viral antigen specific as they only reacted with lysates from infected cells (Fig 8C).

Similar to uninfected (YFP-) GC B cells, the majority of antibodies cloned from infected (YFP+) GC B cells were non-reactive (Fig 8D and 8F), with the remaining antibodies reacting equally to uninfected and infected lysates (Fig 8E and 8G). Importantly, we did not clone any antibodies from infected (YFP+) GC B cells that recognized viral antigen. Additionally, 4 of the 8 antibodies cloned from infected (YFP+) Igλ+ GC B cells expressed IGHV10-1*01, none of which reacted with either viral antigens or lysates from uninfected cells (Fig 8F). In the plasma cell fractions, all cloned antibodies from both uninfected (YFP-) and infected (YFP+) cells were non-reactive (Fig 7H–7J). Of the 16 antibodies cloned from infected (YFP+) Igλ+ plasma cells, 3 expressed IGHV10-1*-1 and 2 expressed IGHV10-3*01. Taken together, these data indicate that the majority of GC B cells and plasma cells generated during MHV68 infection are not viral antigen specific, and that the bias seen in the infected (YFP+) Igλ+ populations does not appear to be driven by antigen selection.

## Discussion

Previous work has demonstrated the MHV68 infection of B cells is not random [11]. The data presented here support this in that MHV68 infected B cells are biased towards cells that express the Igλ light chain together with the IGHV10-1*01 VH gene. While we are not sure why infection is biased towards Igλ expressing cells, it has been shown that KSHV preferentially infects Igλ expressing B cells in vitro [21]. It has also been reported that KSHV infection of primary B cells in vitro induces RAG1/2 gene expression, resulting in receptor editing where Igκ is replaced by Igλ [27]. Furthermore, KHSV infected cells in Multicentric Castleman disease (MCD) are lambda chain restricted [22–24]. However, it is not known if KSHV infected Igλ+ cells are also biased in VH gene usage. While these studies suggest that Igλ bias is a conserved feature of gamma 2 herpesviruses, it is important to note that the work presented here was performed in inbred, genetically identical mice and it is not known if this same bias occurs in outbred strains of mice, which would be more similar to the setting of KSHV infection in humans.

While we were not able to determine the specificity of antibodies cloned from Igλ+ cells that expressed the IGHV10-1*01 VH gene, it is clear that some form of selection is responsible for the bias seen towards cells that express this combination of Ig genes. It has been shown that in C57BL/6 mice, IGHV10-1*01 is expressed in 0.75% of the IgM+ B cell population [28]. If light chain usage in IGHV10-1*01 expressing B cells is stochastic, then only ca. 10% of this population would express Igλ light chain, making this population even rarer. Elucidation of the specificity of these cells will be important for explaining how this population arises. However, if it were merely antigen specificity responsible for expansion of these populations, uninfected cells would also be expected to be biased towards cells expressing this combination of Ig genes–which is not the case.

It is notable that a significant fraction of both infected and uninfected cells appear to be IgM+ memory B cells and IgM+ long-lived plasma cells. IgM memory B cells are capable of re-entering the GC reaction and undergo class switching as well somatic hypermutation [29]. IgM+ long-lived plasma cells can undergo somatic hypermutation in the absence of a GC reaction and are retained in the spleen [30], unlike class-switched long-lived plasma cells which home to the bone marrow. Previous studies have shown that IgM+ memory B cells preferentially enter the GC reaction upon re-challenge, whereas class-switched memory B cells preferentially differentiate into plasma cells [29, 31, 32]. However, others have shown that IgM+ memory B cells preferentially differentiate into plasma cells whereas class switched memory B cells preferentially re-enter the GC reaction [33]. While we do not know what role unswitched memory B cells play during MHV68 infection, the presence of MHV68 infected IgM+ GC B as well as IgM+ plasma cells with somatic hypermutation suggests that unswitched memory cells play an important role in MHV68 biology. The ability of latently infected, GC B cells to re-enter the GC reaction and undergo additional rounds of proliferation would result in an increased pool of infected cells in the absence of virus production. Alternatively, if MHV68 infected GC B cells preferentially differentiate to plasma cells, this may allow for re-seeding of latency reservoirs as differentiation of gammaherpesvirus infected B cells to plasma cells is linked to viral reactivation [8, 34–38].

During primary infection, MHV68 induces a non-specific humoral response that far exceeds the virus specific response [26]. Additionally, MHV68 has been shown to specifically inhibit the virus specific humoral response [39, 40]. Because of this, it is not surprising that the majority of antibodies cloned from uninfected (YFP-) cells were not viral antigen specific. However, MHV68 infects only a small fraction of germinal center B cells [3, 7, 41] and plasma cells [41], so it was not known what contribution these cells make to the overall humoral response. The data presented here suggests that MHV68 infected cells are similar to uninfected cells in that they are largely not viral antigen specific. However, while some viral antigen specific antibodies were cloned from uninfected GC B cells, none were cloned from infected cells, which may be due to the relatively small sample size of cloned antibodies. However, it has been shown that cross-linking the BCR of infected cells along with CD40 stimulation induces viral reactivation [42], so it is possible that infection of virus specific germinal center B cells would result in cell death due to lytic replication.

The lack of somatic hypermutation in a significant percentage of class-switched plasma cells is somewhat surprising. While the majority of both uninfected (YFP-) and infected (YFP+) GC B cells displayed SHM, the majority of uninfected (YFP-) and infected (YFP+) class-switched plasma cells displayed none. Class-switched plasma cells are post-germinal center, and therefore would be expected to have similar or even higher levels of SHM than GC B cells, which have not completed the germinal center reaction and are still capable of undergoing SHM. MHV68 may specifically inhibit SHM in GC B cells destined to become plasma cells, or alternatively may cause these cells to exit the GC reaction prematurely. Whatever the mechanism, inhibition of SHM and affinity maturation would inhibit production of highly specific antibodies, which may be an important aspect of how MHV68 suppresses the specific antibody response. It has been shown that highly specific, broadly neutralizing antibodies to HIV [43], as well as autoantibodies cloned from systemic lupus erythematosus patients [44] and pemphigus vulgaris patients [45], lose specificity upon reversion to their germline sequence. Furthermore, this data suggests that SHM may not be a good indicator to distinguish short-lived, extra-follicular plasma cells from germinal center derived, long-lived plasma cells induced during gammaherpesvirus infection.

In summary, MHV68 infection of B cells is not a random event–there is clearly a bias for infection and/or expansion of Igλ+ IGHV10-1*01 expressing cells, which are a relatively minor population of GC B cells (ca. 2% of GC B cells, as assessed in our analysis of uninfected B cells). Identification of the specificity of antibodies containing this combination of variable genes may provide important insights into the mechanism of this expansion. Additionally, the alterations in both class-switching and SHM during MHV68 infection indicate that these modifications play important roles in establishing life-long infection.

## Materials and methods

### Ethics statement

This study was carried out in strict accordance with the recommendations in the Guide for the Care and Use of Laboratory Animals of the National Institutes of Health. The protocol was approved by the Emory University Institutional Animal Care and Use Committee, and in accordance with established guidelines and policies at Emory University School of Medicine (DAR-2003399-ENTRPR-N).

### Mice, viruses and infections

Female C57Bl/6J mice were purchased from Jackson laboratories (ME) at 6–8 weeks of age, and were infected with MHV68-H2bYFP [3] at 8–12 weeks. For each experiment, additional C57Bl/6J mice were infected with wild type MHV68 (WUMS strain, ATCC VR-1465) to differentiate between auto-fluorescing cells and YFP+ cells. Prior to infection, mice were anesthetized with isofluorane and inoculated intranasally with 1,000 pfu of virus diluted in 20 ul of DMEM. At 18 days post-infection, mice were sacrificed by CO2 asphyxiation and spleens were harvested. To obtain single cell suspensions of splenocytes, spleens were homogenized and filtered through 100 um pore size nylon cell strainers (Becton-Dickinson). Red blood cells were lysed with red blood cell lysis buffer (Sigma).

### Flow cytometry and cell sorting

Single cell suspensions of splenocytes were resuspended in PBS containing 2% fetal bovine serum and stained on ice for 20 minutes. Prior to staining, Fc receptors were blocked with anti-16/32 (BD Biosciences). Antibodies used were BV421 conjugated anti-Igλ (BD Biosciences), PE conjugated anti- Igκ (BD Biosciences), PE-Cy7 conjugated anti-CD95 (BD Biosciences), APC-Cy7 conjugated CD19 (BD Biosciences), PE conjugated CD138 (BD Biosciences), PerCP-Cy5.5 conjugated anti-CD3, CD4 and CD8 (Biolegend), Pac Blue conjugated anti-B220, and E-Fluor 660 conjugated anti-GL7 (eBioscience).

For each infection, a separate group of C57Bl6/J mice were infected in parallel with wild type MHV68. Gates for YFP+ populations were set based on the level of auto-fluorescence detected in the FITC channel from mice infected with wt virus. Single cells were sorted based on YFP and surface marker expression (Table 1). Cells were sorted into 96 well plates containing 4 ul of ice cold 0.5X PBS supplemented with 8U/ul of RNAsin (Promega). After sorting, plates were snap frozen in a dry ice/ethanol bath and stored at -80⁰C.

### Cloning immunoglobulin variable regions from single cells

To synthesize cDNA, 4 ul total RNA from single cells was reverse transcribed using Superscript III reverse transcriptase (Invitrogen) in a final volume of 20 ul according to the manufacturer’s protocol. Heavy and light chain variable regions were then PCR amplified in separate semi-nested (*Igh)* or nested (*Igκ* and *Igλ*) PCR reactions using 4 ul cDNA and pools of primers specific for each heavy and light chain gene family as previously described [25]. Briefly, 5 ul of cDNA was PCR amplified by two rounds of semi-nested (*Igh*) or nested PCR (*Igκ* and *Igλ*). PCR reactions were performed in a final volume of 50 ul containing 500 nM of each primer (Table 2), 100 uM each dNTP, and 1.5 U Taq polymerase (Promega). The first round of PCR was performed at 94°C for 5 minutes followed by 50 cycles of 94°C for 30s, 56°C (*Igh*), 50°C (*Igκ)* or 58°C (*Igλ*) for 30s, and 72°C for 55s followed by a final incubation at 72°C for 5 minutes. The second round of amplification was performed using 5 ul of first round product at 94°C for 5 minutes followed by 50 cycles of 94°C for 30s, 60°C (*Igh*), 45°C (*Igκ)* or 58°C (*Igλ*) for 45s, and 72°C for 55s, followed by a final incubation at 72°C for 5 minutes. PCR products were resolved on a 1.2% agarose gel, purified and sequenced using the second round forward primer. Nucleotide sequences were identified using IgBlast or VBASE2 [46] to identify variable, D and J genes. Once the variable and J genes were identified, primers specific for each gene (Table 3) were designed to clone each gene into expression vectors provided by Hedda Wardemann [25]. These vectors contain the human IgG1, Igκ or Igλ constant regions restriction sites to facilitate cloning; AgeI and SalI for *Igh*, AgeI and BsiWI for *Igκ*, and AgeI and XhoI for *Igλ*.

**Table 2:** 

	Primer name	Primer	Reference
IgH 1st round PCR	5MsVHE	GGGAATTCGAGGTGCAGCTGCAGGAGTCTGG	[53]
	3Cμ outer	AGGGGGCTCTCGCAGGAGACGAGG	[54]
	3Cγ1 outer	GGAAGGTGTGCACACCGCTGGAC	[54]
	3Cγ2c outer	GGAAGGTGTGCACACCACTGGAC	[54]
	3Cγ2b outer	GGAAGGTGTGCACACTGCTGGAC	[54]
	3Cγ3 outer	AGACTGTGCGCACACCGCTGGAC	[54]
IgH 2nd round PCR	5MsVHE	GGGAATTCGAGGTGCAGCTGCAGGAGTCTGG	[53]
	3Cμ inner	AGGGGGAAGACATTTGGGAAGGAC	[54]
	3Cγ1 inner	GCTCAGGGAAATAGCCCTTGAC	[54]
	3Cγ2c inner	GCTCAGGGAAATAACCCTTGAC	[54]
	3Cγ2b inner	ACTCAGGGAAGTAGCCCTTGAC	[54]
	3Cγ3 inner	GCTCAGGGAAGTAGCCTTTGAC	[54]
IgK 1st round PCR	5Vκ_3	TGCTGCTGCTCTGGGTTCCAG	[55]
	5Vκ_4	ATTWTCAGCTTCCTGCTAATC	[55]
	5Vκ_5	TTTTGCTTTTCTGGATTYCAG	[55]
	5Vκ_6	TCGTGTTKCTSTGGTTGTCTG	[55]
	5Vκ_6,8,9	ATGGAATCACAGRCYCWGGT	[55]
	5Vκ_14	TCTTGTTGCTCTGGTTYCCAG	[55]
	5Vκ_19	CAGTTCCTGGGGCTCTTGTTGTTC	[25]
	5Vκ_20	CTCACTAGCTCTTCTCCTC	[25]
	3mCκ	GATGGTGGGAAGATGGATACAGTT	[25]
IgK 2nd round PCR	5mVkappa	GAYATTGTGMTSACMCARWCTMCA	[25]
	3P-mJK01	GCCACCGTACGTTTGATTTCCAGCTTGGTG	[25]
	3P-mJK02	GCCACCGTACGTTTTATTTCCAGCTTGGTC	[25]
	3P-mJK03	GCCACCGTACGTTTTATTTCCAACTTTGTC	[25]
	3P-mJK04	GCCACCGTACGTTTCAGCTCCAGCTTGGTC	[25]
Igl 1st round PCR	Vλ1/2	CAGGCTGTTGTGACTCAG	[25]
	Vλx	CAACTTGTGCTCACTCAG	[25]
	Cλ outer	GTACCATYTGCCTTCCAGKCCACT	[25]
	Cλ inner	CTCYTCAGRGGAAGGTGGRAACA	[25]

**Table 3:** 

IgH primer name	Primer	Reference
VH01	CTGCAACCGGTGTACATTCCCAGGTGCAGCTGCAGCAGCCTGG	[25]
VH02	CTGCAACCGGTGTACATTCCCAGGTGCAGCTGCAGCAGTCTGG	[25]
VH03	CTGCAACCGGTGTACATTCCCAGGTGCAGCTGAAGCAGTCTGG	[25]
VH04	CTGCAACCGGTGTACATTCCCAGGTGCAGCTGAAGGAGTCTGG	[25]
VH05	CTGCAACCGGTGTACATTCCGAGGTGAAGCTGGAGGAGTCTGG	[25]
VH06	CTGCAACCGGTGTACATTCCGAGGTGCAGCTGGTGGAGTCTGG	[25]
VH07	CTGCAACCGGTGTACATTCCGAAGTGCAGCTGTTGGAGACTGG	[25]
VH08	CTGCAACCGGTGTACATTCCGAGGTGCAGCTGCAGCAGTCTGG	[25]
VH09	CTGCAACCGGTGTACATTCCGAGGTGCAGCTGCAGGAGTCTGG	[25]
VH11	CTGCAACCGGTGTACATTCCGAGGTGAAGCTGGTGGAGTCTGG	[25]
VH14	CTGCAACCGGTGTACATTCCGAGTTCCAGCTGCAGCAGTCTGG	[25]
VH15	CTGCAACCGGTGTACATTCCGATGTACAGCTTCAGGAGTCAGG	[25]
VH16	CTGCAACCGGTGTACATTCCGAGGTGCAGCTTGTTGAGTCTGGTGGAGG	[25]
VH22	CTGCAACCGGTGTACATTCCCAGGCTTATCTACAGCAGTCTGG	[25]
VH24	CTGCAACCGGTGTACATTCCCAGGTTCAGCTGCAGCAGTCTG	This work
VH25	CTGCAACCGGTGTACATTCCCAGGTTCAGCTGCAGCAGTCT	This work
VH28	CTGCAACCGGTGTACATTCCCAGGTCCAACTGCAGCAGCC	This work
VH29	CTGCAACCGGTGTACATTCCCAGGTCCAGCTACAGCAGTCT	This work
VH31	CTGCAACCGGTGTACATTCCGAGGTTCAGCTCCAGCAGTCTG	This work
VH32	CTGCAACCGGTGTACATTCCCAGGTCCAGCTGGAGCAGTCT	This work
VH33	CTGCAACCGGTGTACATTCCCAGGTGCAGCTGAAGGAGTCA	This work
VH34	CTGCAACCGGTGTACATTCCCAGGTGCAGGTTCAGCTGCAGCAG	This work
VH35	CTGCAACCGGTGTACATTCCCAGGTGCAGGTCCAGCTGCAGCA	This work
VH36	CTGCAACCGGTGTACATTCCCAGGTGCAGGTCCAGCTGCAGCAGTC	This work
VH37	CTGCAACCGGTGTACATTCCGAAGTGCAGCTGGTGGAGTCT	This work
VH39	CTGCAACCGGTGTACATTCCGAGGTTCAGCTGCAGCAGTCT	This work
JH01	TGCGAAGTCGACGCTGAGGAGACGGTGACCGTGG	[25]
JH02	TGCGAAGTCGACGCTGAGGAGACTGTGAGAGTGG	[25]
JH03	TGCGAAGTCGACGCTGCAGAGACAGTGACCAGAG	[25]
JH04	TGCGAAGTCGACGCTGAGGAGACGGTGACTGAGG	[25]
Igκ primer name	Primer	Reference
VK01	CTGCAACCGGTGTACATTCCAACATTATGATGACACAGTCGCCA	[25]
VK02	CTGCAACCGGTGTACATTCCAACATTGTGCTGACCCAATCTCCA	[25]
VK03	CTGCAACCGGTGTACATTCCCAAATTGTTCTCACCCAGTCTCCA	[25]
VK04	CTGCAACCGGTGTACATTCCCAAATTGTTCTCTCCCAGTCTCCA	[25]
VK05	CTGCAACCGGTGTACATTCCGAAAATGTTCTCACCCAGTCTCCA	[25]
VK06	CTGCAACCGGTGTACATTCCGAAACAACTGTGACCCAGTCTCCA	[25]
VK07	CTGCAACCGGTGTACATTCCGAAATTGTGCTCACTCAGTCTCCA	[25]
VK08	CTGCAACCGGTGTACATTCCGACATCAAGATGACCCAGTCTCCA	[25]
VK09	CTGCAACCGGTGTACATTCCGACATCCAGATGAACCAGTCTCCA	[25]
VK10	CTGCAACCGGTGTACATTCCGACATCCAGATGACTCAGTCTCCA	[25]
VK11	CTGCAACCGGTGTACATTCCGACATTGTGATGACTCAGTCTC	[25]
VK12	CTGCAACCGGTGTACATTCCGACATTGTGATGTCACAGTCTCCA	[25]
VK13	CTGCAACCGGTGTACATTCCGACATTGTGCTGACCCAATCTCCA	[25]
VK14	CTGCAACCGGTGTACATTCCGATATCCAGATGACACAGACTACA	[25]
VK15	CTGCAACCGGTGTACATTCCGATGTTGTGATGACCCAAACTCCA	[25]
VK16	CTGCAACCGGTGTACATTCCGAAATCCAGATGACCCAGTCTCCA	[25]
VK17	CTGCAACCGGTGTACATTCCGACATCCAGATGACACAATCTTCA	[25]
VK18	CTGCAACCGGTGTACATTCCGACATCCAGATGACCCAGTCTCCA	[25]
VK19	CTGCAACCGGTGTACATTCCGACATCCTGATGACCCAATCTCCA	[25]
VK20	CTGCAACCGGTGTACATTCCGACATTGTGCTCACCCAATCTCC	[25]
VK21	CTGCAACCGGTGTACATTCCGATGTTGTGGTGACTCAAACTCCA	[25]
VK22	CTGCAACCGGTGTACATTCCAACATTGTAATGACCCAATCTCCC	[25]
VK23	CTGCAACCGGTGTACATTCCGATGTTTTGATGACCCAAACTCCA	[25]
VK24	CTGCAACCGGTGTACATTCCGATATTGTGATGACTCAGGCTGCA	[25]
VK25	CTGCAACCGGTGTACATTCCGACATCCAGATGATTCAGTCTCCA	[25]
VK26	CTGCAACCGGTGTACATTCCGACATCTTGCTGACTCAGTCTCCA	[25]
VK27	CTGCAACCGGTGTACATTCCGATGTCCAGATGATTCAGTCTCCA	[25]
VK28	CTGCAACCGGTGTACATTCCGATGTCCAGATAACCCAGTCTCCA	[25]
VK29	CTGCAACCGGTGTACATTCCCAAATTGTTCTCACCCAGTCTCC	This work
VK30	CTGCAACCGGTGTACATTCCGACATCCAGATGACACAGTCTCC	This work
VK31	CTGCAACCGGTGTACATTCCGACATTGTGATGACACAGTCTCC	This work
VK35	CTGCAACCGGTGTACATTCCGACATCCAGATGACCCAGTCTC	This work
VK36	CTGCAACCGGTGTACATTCCGATGTTGTTCTGACCCAAACTCC	This work
VK37	CTGCAACCGGTGTACATTCCAACATTATGATGTTGTTCTGACCCAAACTCC	This work
VK38	CTGCAACCGGTGTACATTCCAACATTATGACATTGTGATGACCCAGTCTCA	This work
VK39	CTGCAACCGGTGTACATTCCGAAATGGTTCTCACCCAGTCTCC	This work
JK01	GCCACCGTACGTTTGATTTCCAGCTTGGTG	[25]
JK02	GCCACCGTACGTTTTATTTCCAGCTTGGTC	[25]
JK03	GCCACCGTACGTTTTATTTCCAACTTTGTC	[25]
JK04	GCCACCGTACGTTTCAGCTCCAGCTTGGTC	[25]
Igλ primer name	Primer	Reference
VL01	CTGCTACCGGTTCCTGGGCCCAGGCTGTTGTGACTCAG	[25]
VL02	CTGCTACCGGTTCCTGGGCCCAACTTGTGCTCACTCAG	[25]
JL01	TTGGGCTGGCCAAGGACAGTCAGTTTGGTTCC	Modified from [25]
JL02	TTGGGCTGGCCAAGGACAGTGACCTTGGTTCC	Modified from [25]
JL03	TTGGGCTGGCCAAGGACAGTCAATCTGGTTCC	Modified from [25]

### Expression and purification of recombinant antibodies

For transient transfections, HEK 293T cells (ATCC) were grown in Dulbecco’s modification of Eagle medium (DMEM) supplemented with 10% fetal bovine serum, 2 mM L-glutamine, and 100 U of penicillin and 100 mg of streptomycin per ml. DMEM supplemented with 10% FBS. To express recombinant antibodies, 7.5 ug of each matched pairs of heavy and light chain expression vectors cloned from a single cell were co-transfected into HEK 293T cells in 10cm dishes using Mirus Transit-LT-1 transfection reagent (Mirus). The following day, supernatants were removed and replaced with 10 ml of EX-CELL 293 serum free media (SAFC Biosciences). Supernatants containing expressed antibodies were harvested 5–6 days later and cleared of cell debris by centrifugation at 1,000g for 10 minutes. Antibodies were purified by addition of 25ul of Protein G agarose beads (Pierce) and incubated with rotation overnight at 4°C. The beads were then pelleted by centrifugation at 800g for 5 minutes, resuspended in 200 ul PBS and transferred to a chromatography spin column (Bio-Rad) equilibrated with PBS. The beads were washed 2 times with PBS, then eluted in 3 fractions of 200 ul each with 0.1M glycine (pH3.0) into tubes containing 20 ul of 1M Tris (pH8.0). Sodium azide was then added to a final concentration of 0.1%.

### ELISA

Antibody concentrations were determined by sandwich ELISA as previously described [25]. Briefly, high binding capacity 96 well plates (Nunc) were coated with 50 ul per well of anti-human kappa or anti-human lambda (Rockland) at a concentration of 2ug/ml in PBS, and incubated overnight at room temperature. Plates were washed 3 times with diH_2_O and then incubated for 1 hour with 200 ul of blocking buffer (0.05% Tween-20 in PBS with 2mM EDTA). Purified human IgG1 kappa and IgG1 lambda (Sigma) were used as standards, and consisted of 2-fold serial dilutions starting at 25 ng/mL. Purified antibodies were serially diluted 2-fold, starting at a 500-old dilution. 50 ul of each dilution was transferred to the ELISA plate and incubated at room temperature for 2 hours. Plates were then washed with 3 times blocking buffer and incubated for 2 hours with 50 ul of horseradish peroxidase (HRP) conjugated goat anti-human IgG (Jackson) at 0.8 ug/mL in blocking buffer for 2 hours. Plates were washed 3 times with water and then developed with TMB substrate (BD Biosciences) according to the manufacturer’s instructions. Optical density was read at 450nm with 570nm correction and antibody concentration was determined using Gen5 Software.

Reactivity to viral antigens was tested by ELISA using lysates from NIH3T12 cells (ATCC) infected with MHV68H2bYFP or uninfected NIH3T12 cells as capture antigen. Ninety-six well plates were coated with sonicated lysates at a concentration of 5 ug/mL, and incubated for 48 hours at 4°C. Four-fold dilutions of purified antibodies, starting at 1 ug/mL, were incubated for 2 hours at room temperature. Binding was detected with horseradish peroxidase (HRP) conjugated goat anti-human IgG (Jackson) and TMB (BD Biosciences) was used as a substrate according to the manufacturer’s protocol. Optical density was read at 450nm.

### RNA-seq and data analysis

Splenocytes were sorted at day 18 post-infection from C57Bl/6J mice infected intranasally with 1,000 pfu of MHV68-H2bYFP. Prior to extraction, 5 ul of a 1:40,000 dilution of External RNA Controls Consortium (ERCC) control RNA was added to each sample, and total RNA was isolated using the Quick-RNA MicroPrep Kit (Zymo Research) and used as input for the SMART-seq v3 cDNA synthesis kit (Takara) using 10 cycles of PCR amplification. 400 pg of cDNA was used as input for the NexteraXT kit (Illumina) using 10 cycles of PCR amplification. Final libraries were quantitated by qPCR and sized distribution determined by Bioanalyzer prior to pooling and sequencing on a HiSeq2500 using 50bp PE chemistry. Raw fastq files were mapped to a combined build of the ERCC[47], MHV68 [48], and mm9 genome using Tophat2 v2.0.13[49] with the default parameters and the UCSC KnownGene reference transcriptome[50]. Duplicate reads were removed with PICARD v1.127 (http://broadinstitute.github.io/picard/). BCR segment locations were downloaded from the IMGT database[51] and sequencing coverage for all segments was summarized using custom R/Bioconductor scripts and the GeonomicRanges v1.22.4[52] package and normalized to fragments per kilobase per million reads (FPKM).

### Statistical analysis

Graphpad Prism software was used to calculate p values differences in light chain distribution and in the level of somatic hypermutation. Chi square test was used to calculate p values for light chain distribution and Mann-Whitney U test was used to calculate p values for differences in the level of somatic hypermutation. p values to calculate differences in isotype usage among total populations was calculated by Fisher’s exact test with Bonferroni correction for multiple comparisons, and p values to determine which isotype within each population was significantly different was calculated by Fisher’s exact test of two proportions with Holm’s correction. p values to calculate differences in isotype usage between Igκ+ and Igλ+ populations was calculated by Fisher’s exact test with Holm’s correction. In making all of these comparisons the overall family-wise error rate was held at 5%.

## Supporting information

S1 Table Single cell PCR analyses carried out on the indicated populations of GC B cells and plasma cells. Shown is the total number of PCR reactions carried out, the number of PCR reactions that yielded the indicated product, and the total number of unique sequences obtained for each population. These analyses are summarized in the pie charts shown in Fig 1.
(DOCX)
