# Supplementary material for: Murine gammaherpesvirus infection is skewed toward Igλ+ B cells expressing a specific heavy chain V-segment
Source: PLoS Pathog. 2020 Apr 30;16(4):e1008438. doi: 10.1371/journal.ppat.1008438 (PMC7217478; doi:10.1371/journal.ppat.1008438)
Supplement: S1 Table — Shown is the total number of PCR reactions carried out, the number of PCR reactions that yielded the indicated product, and the total number of unique sequences obtained for each population. These analyses are summarized in the pie charts shown in Fig 1. (DOCX) [file ppat.1008438.s001.docx]

**Table S1**

|  | YFP- GC B cells | YFP+ GC B cells | YFP- plasma cells | YFP+ plasma cells |
| --- | --- | --- | --- | --- |
| Total PCR reactions | 480 | 336 | 376 | 402 |
| VH+ reactions^a^ | 91 | 117 | 148 | 167 |
| Vκ+ reactions^a^ | 186 | 130 | 157 | 125 |
| Vλ+ reactions^a^ | 31 | 134 | 44 | 175 |
| VH sequences^b^ | 62 | 86 | 122 | 136 |
| Vκ sequences^b^ | 45 | 41 | 97 | 50 |
| Vλ sequences^b^ | 6 | 52 | 20 | 84 |

a- number of PCR reactions that yielded a product for the indicated populations

b- number of sequences obtained for each of the indicated populations
